# Changes in the Welfare of an Injured Working Farm Dog Assessed Using the Five Domains Model

**DOI:** 10.3390/ani6090058

**Published:** 2016-09-21

**Authors:** Katherine E. Littlewood, David J. Mellor

**Affiliations:** Animal Welfare Science and Bioethics Centre, Institute of Veterinary, Animal and Biomedical Sciences, Massey University, Palmerston North 4442, New Zealand; K.Littlewood@massey.ac.nz

**Keywords:** animal welfare assessment, working dog welfare, leg injury, veterinary evaluation, amputation, rehoming as amputee, negative experiences, positive experiences, quality of life

## Abstract

**Simple Summary:**

The Five Domains Model is now increasingly used to assess the welfare status of a wide range of species in markedly different circumstances. Particular strengths are that the Model facilitates structured, systematic and comprehensive evaluations of animals’ negative and positive mental experiences, the overall balance of which underlies their welfare status or quality of life. Importantly, the Model also clarifies the specific internal and external factors that give rise to those experiences. The welfare evaluation published here is the first to use the most up-to-date version of the Model, and stands as a detailed example that may assist others undertaking such welfare evaluations in other species and contexts. Moreover, it is the first such evaluation of a companion animal. It employs a fictitious scenario involving a working farm dog before, during and after it sustains a serious hind leg injury requiring amputation and its subsequent rehoming as a pet. A wide range of negative and positive experiences are graded, interactions between them are revealed, and the balance between negative and positive states at different stages of the scenario is described. Such Model evaluations can highlight current practices that merit re-evaluation. More generally, when major welfare issues are identified, use of the Model could enhance expert witness participation in related prosecutions by highlighting scientifically supported connections between indicative physical/functional states and behaviours and their associated negative experiences in ill-treated animals. Five Domains Model evaluations can also facilitate quality of life assessments and end-of-life decisions.

**Abstract:**

The present structured, systematic and comprehensive welfare evaluation of an injured working farm dog using the Five Domains Model is of interest in its own right. It is also an example for others wanting to apply the Model to welfare evaluations in different species and contexts. Six stages of a fictitious scenario involving the dog are considered: (1) its on-farm circumstances before one hind leg is injured; (2) its entanglement in barbed wire, cutting it free and transporting it to a veterinary clinic; (3) the initial veterinary examination and overnight stay; (4) amputation of the limb and immediate post-operative recovery; (5) its first four weeks after rehoming to a lifestyle block; and (6) its subsequent life as an amputee and pet. Not all features of the scenario represent average-to-good practice; indeed, some have been selected to indicate poor practice. It is shown how the Model can draw attention to areas of animal welfare concern and, importantly, to how welfare enhancement may be impeded or facilitated. Also illustrated is how the welfare implications of a sequence of events can be traced and evaluated, and, in relation to specific situations, how the degrees of welfare compromise and enhancement may be graded. In addition, the choice of a companion animal, contrasting its welfare status as a working dog and pet, and considering its treatment in a veterinary clinical setting, help to highlight various welfare impacts of some practices. By focussing attention on welfare problems, the Model can guide the implementation of remedies, including ways of promoting positive welfare states. Finally, wider applications of the Five Domains Model are noted: by enabling both negative and positive welfare-relevant experiences to be graded, the Model can be applied to quality of life assessments and end-of-life decisions and, with particular regard to negative experiences, the Model can also help to strengthen expert witness testimony during prosecutions for serious ill treatment of animals.

## 1. Introduction

This paper provides an example of how animal welfare can be assessed in a structured, systematic and comprehensive fashion using the Five Domains Model. The example addresses a fictitious sequence of events involving a working farm dog that sustained a leg injury requiring amputation and subsequent rehoming to a lifestyle block as a pet. This analysis will be of interest to companion animal veterinarians and pet owners, as well as to others with an interest in confident and competent animal welfare assessments, including conscientious animal care personnel, members of animal welfare NGOs, those with an interest in rehoming pets, farmers and farm staff, and animal-based scientists.

The Five Domains Model used here is a recently updated version that facilitates the characterisation and grading of different forms of animal welfare compromise linked to particular negative experiences and different forms of welfare enhancement linked to specified positive experiences [1]. The Model therefore incorporates the increasing recognition that for welfare management to be regarded as ‘acceptable’, the minimisation of negative welfare states must be accompanied by the promotion of positive ones [2,3,4,5,6,7,8]. Moreover, no other assessment system currently available appears to emphasise as strongly the value of considering the wide range of potential negative and positive experiences that may arise in the diverse situations in which animals find themselves [1,9,10,11].

Previous versions of the Five Domains Model focussed only on welfare compromise. For example, they were used to assess the negative impacts of scientific procedures [9,12,13] and lethal or non-lethal methods for controlling vertebrate pests [10,14,15,16], as well as welfare problems in farm livestock [17,18]. Recently, reflecting the current impetus to also include positive experiences in welfare assessments, the World Association of Zoos and Aquariums incorporated the latest version of the Model [1] into its global animal welfare strategy [19].

The paper begins by outlining the fictitious scenario involving a working farm dog and then describes briefly how welfare assessments are conducted using the Five Domains Model. A detailed Model-based welfare evaluation of the dog before, during and after it sustains a major injury to a hind leg is then undertaken in order to show readers how to use the Model. It provides a thorough description of the origins and likely character of the negative or positive subjective experiences the dog may have had, and also provides detailed examples of the grading of these experiences in terms of welfare compromise or enhancement. The paper ends with a brief discussion of a range of general and specific points raised by the evaluation.

## 2. The Scenario

The fictitious scenario describes the circumstances of a working farm dog during a sequence of events that included a serious hind leg injury leading to amputation and the need to rehome it as a pet. Six stages have been described: (1) the circumstances of the dog before the traumatic injury; (2) the features of the injury caused by barbed wire, the farmer freeing the dog and taking it for veterinary treatment; (3) the initial veterinary examination and overnight stay; (4) the amputation and immediate post-operative recovery; (5) transport of the dog to and its first four weeks in its new home on a lifestyle block; and (6) its subsequent life as an amputee and pet. See Table 1 for details of these stages. Note that Section 4 provides extensive additional information on each stage of the scenario in the detailed descriptions of the dog’s internal physical/functional states and the likely impacts on the dog of its external circumstances. Note also that not all features of the scenario represent average-to-good practice; indeed, some have been selected to indicate poor practice.

## 3. Five Domains Animal Welfare Assessment

**Figure 1:**
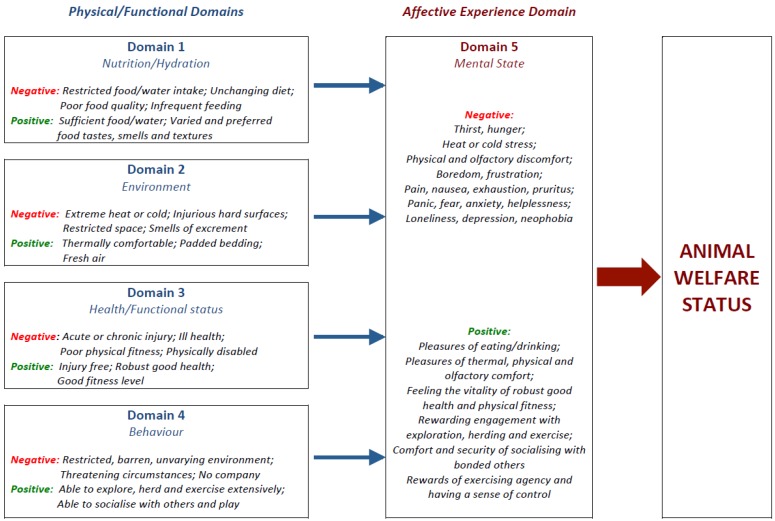
An abbreviated schema of the Five Domains Model (adapted from [20]) showing negative and positive physical/functional states or situations (Domains 1–4) and their associated negative and positive experiences or affects (Domain 5) relevant to the working farm dog scenario. Taken together, these mental experiences represent the overall Welfare Status of the animal, and the balance between the positive and negative experiences its Quality of Life. A more detailed general schema of the Model is available elsewhere [1].

The Five Domains Model is not a definition of animal welfare. Nor is it intended to be an accurate anatomical or physiological representation of the body [12]. Rather, it is a facilitatory assessment and grading device based on current scientific understanding of animal welfare; it also provides numerous examples of welfare compromise and enhancement potentially applicable to a wide range of mammals [1]. The Model recognises that an animal’s welfare state reflects what the animal itself experiences subjectively, i.e., its affective experiences or affects [3,11,18,21]. Moreover, it is structured to differentiate between the internal and external physical/functional conditions or circumstances that give rise to different affects and specifies a wide range of them, both negative and positive, to aid in their cautious evaluation. Domains 1–4 (“nutrition”, “environment”, “health” and “behavior”) focus attention on the animal’s internal and external conditions or circumstances, whereas Domain 5 (“mental state”) focuses attention on all of the affective experiences elicited by the conditions or circumstances evaluated in Domains 1–4 (Figure 1). The Model therefore incorporates the ‘biological functioning’ and ‘affective state’ orientations towards animal welfare identified by Fraser et al. [22].

The first step in each analysis is therefore to identify the origins (internal or external), valence (negative or positive), and character of significant affective experiences in each situation. A grade is then assigned to their welfare impact on the animal. Numerous specific examples of grading are provided in Section 4. As explained elsewhere [1], there are different approaches to grading depending on whether the experiences are negative (contributing to compromise) or positive (contributing to enhancement) (Table 2). Note that numerical grading using the Model was explicitly rejected in order to avoid facile, non-reflective averaging of ‘scores’ as a substitute for considered judgement and to avoid implying a degree of precision that is not achievable [12,13,23,24]. Moreover, scientifically informed best judgement is emphasised as a key element in the use of the Model.

The degree of *compromise* in each domain is graded on a five-point scale ranging from A (none) to E (very severe) [12,18]. Key general elements in assigning these grades to Domains 1–4 include the observed severity of functional disruptions and/or negative impacts on behaviour, the duration of these disruptions and/or impacts, their likely reversibility using mitigating husbandry or therapeutic interventions, and, at the extreme, the need for euthanasia [12,18,25]. More specific principles that characterise and differentiate between different grades for welfare compromise over the full range from A to E in each domain are outlined elsewhere [18]. There are numerous well-validated behavioural, anatomical, physiological, neurological, pathological and clinical diagnostic indices of such disruptions or impacts in Domains 1–4 (e.g., [26,27,28]). Also well validated is their alignment with particular affects (e.g., [3,29,30,31,32]), which in the context of the Model are assigned to Domain 5. Finally, the overall grade of welfare compromise is derived by cautious assessment of the accumulated impacts of all of the negative affects assigned to Domain 5, which represents the animal’s mental state [1,12].

Compromise associated with Domains 1–3 relates mainly to disruptions or imbalances in the animal’s *internal* physical/functional states, and these give rise to negative affects and related behaviours that are critical to the animal’s survival [7]. These *survival-critical affects*, assigned to Domain 5, include breathlessness, thirst, hunger, pain, nausea, dizziness, sickness and weakness [1,32]. On the other hand, Domain 4 mainly focuses on brain processing that contributes to the animals’ perceptions of their *external* circumstances, which, if threatening, cramped, barren and/or devoid of the company of other animals, may give rise to a range of other negative affects. Also assigned to Domain 5, these affects include anxiety, fear, panic, frustration, anger, helplessness, loneliness, boredom and depression [1]. Note finally that neutralising the *internally generated* negative affects does not, in and of itself, result in positive affective outcomes, but improving the *external circumstances* of animals can lead to previously experienced negative affects being replaced with positive ones (see below) [1,7,33].

Animal welfare *enhancement* may be understood in terms of “positive affective engagement” [1]. This concept is intended to capture the experiences animals may have when they actively respond to motivations to undertake behaviours they find rewarding, and it potentially incorporates all of the associated affects that are positive [7,8]. Thus, it includes the genetically pre-programmed, or learned, affectively positive impulses to engage in rewarding behaviours, and it also includes positive affects related to anticipation, goal achievement and memory of success [2,30,31,34]. A key element is the extent to which animals are able to exercise “agency”, i.e., whether they can engage in voluntary, self-generated and goal-directed behaviours [35] that are aligned with a general sense of being in control [36].

The welfare *enhancement* scale has four tiers (0, +, ++, +++) (Table 2) and is used to grade the extent to which animals are likely to have positive experiences, all of which are accumulated into Domain 5 [1]. The scale is applied in two ways. The first application captures positive affects generated by the activities or circumstances of survival-critical significance considered in Domains 1–3. For example, in Domain 1, water consumption may generate the wetting/quenching pleasures of drinking, and may accompany and, when anticipated, may reinforce thirst as the major motivation to drink [7,8]. Also, pleasurable smells, tastes and textures of particular foods, when anticipated and experienced, might lead to their selection during hunger-motivated feeding [7,8]. In Domain 2, particular physical circumstances enable animals to have the positive experiences of thermal, physical, respiratory, olfactory, auditory and visual comfort. And finally, in Domain 3 clinical and husbandry interventions can promote the comfort and vitality of good health, high functional capacity and physical fitness [1].

The second application of the welfare *enhancement* scale (0, +, ++, +++) focuses on Domain 4. It has three elements: the identification of the number of *opportunities* available for animals to engage in rewarding behaviours; the animal’s actual *utilisation* of those opportunities; and the overall extent of welfare *enhancement* [1]. In this application, Domain 4 (behaviour) is employed to focus attention on opportunities and their utilization, and Domain 5 (mental state) focuses on the likely associated positive affects. The key to enhancement is the actual use of the available behavioural opportunities [7,8,37]. Thus, enhancement is graded at the same level as that assigned to the number of opportunities that are utilised, and this is taken to represent the overall extent of positive affective engagement cautiously inferred from the observed behaviours [1]. Behavioural, physiological and neuroscience evidence supports this approach (e.g., [3,29,30,31,38]). Such behaviours may include environment-focussed exploration and food acquisition activities as well as animal-to-animal interactive activities such as bonding and bond affirmation, maternal, paternal or group care of young, play behaviour and sexual activity [7,8]. In general terms, the associated positive affects are considered likely to include various forms of comfort, pleasure, interest, confidence and a sense of control [3,4,11,39].

It is apparent that grading welfare compromise first and then enhancement would provide a more comprehensive assessment of the various factors that contribute to an animal’s welfare state at any one time. It would also help to clarify whether or not the intensity and duration of *internally generated* negative affects may significantly demotivate or preclude animals from utilising *externally focussed* behavioural opportunities that they would usually find rewarding [2,38,40,41]. The combined assessment and grading of compromise and enhancement is represented as a composite symbol (Table 2), which covers the range from *very severe* compromise and *no* enhancement, represented as “E/0”, to *no* compromise and *high-level* enhancement, denoted as “A/+++”. This capacity to cautiously grade both negative and positive welfare-related experiences using the Five Domains Model has obvious relevance to quality of life assessments [1,6,11,38,42].

## 4. Welfare Impacts of a Traumatic Injury in a Farm Dog

It is recommended that readers review the details of each stage of the scenario outlined in Table 1 before proceeding to the animal welfare analysis provided below for that stage. Note that the internal states and external circumstances (Domains 1–4) presented for each stage have been selected to highlight a range of features for assigning an overall animal welfare grade (Domain 5). These features are presented as observed facts and provide considerable additional information about the scenario. Accordingly, extensive details are provided for each step of the analysis for each stage of the scenario. Also, the stage-by-stage accumulation of details that underlie the assignment of grades within each domain, considered as a whole for each domain, further clarifies the bases for differentiating between grades over the ranges covered by this scenario. The overall purpose is to show how the presence of specific negative or positive affects may be inferred and how the welfare impacts of those affects may be graded. Finally, note that Table 3 summarises the grades assigned for compromise and/or enhancement in each domain for all six stages of the scenario.

### 4.1. Stage 1: Prior to the Traumatic Injury

This section summarises Jess’s likely experiences whilst working on the sheep farm, noting that the affects of animal welfare significance are likely to have shown daily and seasonal variations. Her *overall* welfare state was graded “C_1_/+” during this stage, representing *mild* compromise (C_1_) and *low-level* enhancement (+). The breakdown of this grading for each of the five domains is outlined below. Note that some features of Jess’s daily welfare management would be below standards regarded as acceptable by many farmers.

#### Domain 1: Nutrition

Jess was fed small amounts of moderate quality food, once daily, providing little variety, and of insufficient quantity and energy content to meet her requirements as an active working dog. Her daily energy intake was estimated to be below the 2–3 times maintenance level required for an active working dog [43,44]. Her poor body condition and below average muscle mass reflected this. Her water intake was judged to be adequate during winter, but due to intermittent filling of her water bowl, she likely had bouts of dehydration when confined in her kennel in summer. As these food and/or water restrictions caused significant long-term effects on her physiological state and body condition, she was graded ‘C_1_’, *mild* compromise in Domain 1 (Table 2; [18]). This accords with the sub-optimal lifetime performance that has been suggested for active sheep dogs fed below maintenance levels [45]. 

#### Domain 2: Environment

Prior to her traumatic injury, Jess occupied two contrasting physical environments: the open spaces of the farm when working during daylight hours, and the markedly restricted space of her kennel and small run every night and when not working. Annually she spent an average of 30% of each day working and 70% confined. When working she exercised widely over variable ground (although under direction) and had access to fresh air and interesting sights, sounds and smells. In both environments she was intermittently exposed to thermal extremes in summer/winter, but with very limited capacity to behaviourally mitigate their effects in her kennel. In addition, she likely experienced muscle tension and stiffness both during and immediately after intense work, and when kennelled for long periods. Close confinement in her kennel also exposed her to developing low-grade pressure sores due to the wooden floors, and she would have experienced mildly unpleasant odours and pollutants from her own and other dogs’ excrement when not cleared away promptly. As physiological coping strategies associated with these physical challenges would for the most part have elicited normal adaptive responses (e.g., heat production through shivering in response to cold exposure), physical/functional compromise in Domain 2 was graded “B”, i.e., *low* (Table 2; [18]).

#### Domain 3: Health

As a young, fit and active working dog Jess was in good health. Her annual worm treatments maintained worm burdens at subclinical levels, which, if they compromised digestive processes of relevance to Domain 1, did so to an unknown extent. As a result of inadequate annual treatment, flea infestation was recognised as a problem during warmer months, causing episodes of moderate pruritus and so a moderate impact on Jess’s experience. Some impact would be expected because sleep disturbances linked to marked pruritus have been observed in dogs with atopic dermatitis [46] and treatment improved their quality of life [47]. As a physically fit, uninjured and largely healthy animal with intermittent, but minor, health challenges, Jess was graded “B”, *low* compromise, for Domain 3 (Table 2). Her body condition score has already been considered via Domain 1, and so does not need to be re-evaluated here [17,18].

#### Domain 4: Behaviour

The two physical environments Jess occupied had markedly different impacts on her opportunities to exercise agency, i.e., her capacity to engage in voluntary, self-generated and goal-directed behaviours that would give her a general sense of being in control [35,36]. These opportunities were substantially curtailed during confinement in her kennel and run for 70% of the time, and much less restricted when she was working on the farm.

The grading of behavioural *compromise* in Domain 4 depended largely on the time Jess spent in her kennel. Its barrenness, the limits it imposed on her thermoregulatory and urination/defecation behaviours, its constraints on interactive activity with other dogs and the farmer, and the virtual impossibility of vigorous exercise, taken together represented significant imposts on Jess. These restrictions applied for long periods and probably led to the repetitive pacing and excessive barking which Jess exhibited [42] and which are well recognised behavioural vices or stereotypies that potentially act to ameliorate such compromise [48,49]. Conversely, the kennel may have provided security and opportunities for rest after long days of active farm work. These long-term restrictions on behavioural expression, if unrelieved by periods of farm work, would have been graded “D_2_”, *severe,* on the compromise scale for Domain 4 (Table 2). However, because Jess could engage in wide ranging behaviours whilst out working on the farm for 30% of the time, i.e., equivalent to about 60% of daylight hours, they were graded “C_1_”, representing *mild* compromise (Table 2; [1,18]).

In contrast, the grading of behavioural *enhancement* in Domain 4 was determined primarily by the length of time Jess spent outside her kennel on the farm where she had greater opportunities to take behavioural initiatives in exercising agency while working. However, despite the rich diversity of this unconfined environment, she could not experience this in an unconstrained way. During periods of work she was largely restricted to following the commands of the farmer rather than being able to roam freely. Nevertheless, she was strongly motivated to follow these commands due to her bond with the farmer and bond affirming interactions with him when she performed well [7]. Likewise, she engaged in some bond affirming activities with the other dogs during this work. Finally, her highly focussed predatory-like stalking and rounding up activities during herding would have enabled Jess to express these genetically pre-programmed or learned behaviours [7,8]. Accordingly, during her farm work Jess utilised mid-level opportunities to engage in rewarding behaviours, which were graded “++” on this scale for Domain 4 [1]. However, because of the marked constraints on behavioural opportunities during confinement in her kennel for the rest of the time, the overall grade for behavioural enhancement was reduced to “+”, low-level (Table 2).

#### Domain 5: Mental State—the Affective Outcomes

As noted above, this domain is the ultimate focus of the welfare assessments for each stage, because the overall grades for compromise and enhancement are decided after cautiously evaluating the accumulated impacts of all negative and all positive affects Jess was deemed likely to have experienced.

*Nutrition.* Jess was probably persistently hungry, indicated by her poor body condition that included a low muscle mass, and supported later by her ready consumption of the food offered at the veterinary clinic, despite her injuries and potential emetic effects of morphine (Stage 3). She also likely experienced periods of moderate thirst due to intermittent water supply in hot weather. Transient low-level welfare enhancement may have occurred via masticatory pleasure associated with brief daily periods of food consumption, but, overall, this was considered likely to have only had a marginal positive impact in this domain [7]. Thus, the overall affective impact in Domain 1 was graded “C_1_/0”, representing *mild* compromise with no enhancement (Table 2; [1,18]).

*Environment.* Some of the physical and meteorological features of her kennel and outdoor working conditions on the farm would have caused periods of physical discomfort due to imposed inactivity/activity and/or exposure to thermal extremes. Exposure to noxious smells of her own and other dogs’ excrement whilst in her kennel was likely balanced by access to interesting odours out on the farm. Likewise, when temperatures were not extreme, Jess probably experienced periods of thermal comfort, which were judged on average to have approximately counterbalanced the thermal discomfort she experienced at other times. Thus, the overall affective impact of her physical environment assessed in Domain 2 was graded “B/0”, i.e., low compromise with no enhancement (Table 2; [1,18]).

*Health.* Jess would likely have experienced the robust vitality of being physically fit, uninjured and largely healthy with only intermittent and minor health challenges. Accordingly, the overall affective impact in Domain 3 was graded as “B/++”, i.e., low compromise with mid-level enhancement (Table 2; [1,18]).

*Behaviour.* The negative affects experienced during periods of enforced inactivity and constraints on exploration, feeding behaviour, bonding/bond affirmation and play behaviour imposed by confinement in her kennel are likely to have included frustration, boredom, helplessness and possibly loneliness [7,8]. Considered separately, these *marked* negative affective consequences of kennelling were graded “D_1_” on the compromise scale. However, as Jess was kennelled for 70% of the time, which included 40% of daylight hours, and was assessed as having positive experiences during her farm work (see below), which occupied 60% of daylight hours, the *overall* negative impact of kennelling was judged to be lower, i.e., *mild*. Thus, compromise in the kennel was graded “C_1_” (Table 2; [1,18]).

The positive affects Jess would have experienced during periods of work on the farm would have been associated with her greater freedom of movement and capacity to exercise agency. They likely included energised expression of her highly focussed genetically pre-programmed or learnt herding behaviour [7,8], bond affirming rewards of being stroked and praised by the farmer for work well done, alert exploration of interesting odours, sights and sounds encountered when moving to and from work paddocks, as well as companionable contact, perhaps occasionally including pleasurable play, with other dogs and the farmer. Of course, these positives may at times have been tempered by significant physical tiredness or exhaustion. Considered separately, therefore, these positive affective consequences of farm work were graded “++”, *mid-level*, on the enhancement scale. However, taking the negative impacts of kennelling between periods of farm work into consideration (Table 2), overall enhancement was graded “+”, *low-level* (Table 2; [1].

*Affective contributions to the overall welfare grade.* In terms of contributory affective experiences, nutrition was graded “C_1_/0”, environment “B/0”, health “B/++” and behaviour “C_1_/+”. It was cautiously concluding that, taken together, the negative affects of hunger, thirst, physical discomfort, pruritus, frustration, boredom and helplessness, offset by rewarding experiences associated with good health, physical fitness and engaged expression of positively motivated herding, exploratory and bond affirming behaviours, likely had the greatest impacts on Jess’s welfare compromise and enhancement, respectively. Thus, overall, her mental state prior to the traumatic injury was graded “C_1_/+”, i.e., *mild* compromise with *low-level* enhancement (Table 3).

### 4.2. Stage 2: The Traumatic Injury

This stage includes entanglement in barbed wire, being cut free and then transported across the farm and by road to the veterinary clinic (Table 1). As detailed below, Jess’s overall affective experience, representing her welfare status, was graded “D_2_/0” during this stage, i.e., *severe* compromise with *no* enhancement.

#### Domain 1: Nutrition

Jess’s overall nutritional status was similar to that described in stage 1. Note that the barbed wire entanglement likely occurred about 20 h after her last meal, and that before becoming entangled she probably had limited access to water and none afterwards; accordingly, nutritional compromise for this stage was graded “C_2_”, *moderate*, and not “C_1_”, *mild* as for stage 1 (Table 2; [18]).

#### Domain 2: Environment

During the late afternoon in mid-May at an outdoor air temperature of 12 °C to 13 °C, with little wind and no rain, Jess would have experienced minor cold exposure. The *low* physical/functional impact of her outdoor environment whilst entangled and when transported back to the farmhouse was therefore graded “B” (Table 2; [18]). This grade was also assigned to her transport to the veterinary clinic. This is because the warmer conditions in the back of the vehicle were somewhat offset by the uncomfortable surfaces and the uneven road making it difficult for Jess to gain purchase and maintain herself in one position.

#### Domain 3: Health

The acute hind limb injury from the barbed wire, made worse by fruitless struggling to escape, involved a full-thickness large laceration and complete severance of the common calcanean tendon (Table 1). Further struggling, exacerbating her injury, occurred whilst the farmer cut her free from the wire. Jess shifted position on the farm bike and later in the back of the utility vehicle in response to the uneven track and road surfaces whilst being transported from the back of the farm and then to the veterinary clinic in town. The associated *marked* physical/functional compromise was assigned an overall grade of “D_1_” (Table 2; [18]).

#### Domain 4: Behaviour

Whilst entangled in the barbed wire Jess’s behaviour was markedly restricted. Her initial escape attempts, which were no doubt vigorous, made her entanglement worse and exacerbated her leg injuries, as did her struggling when the farmer approached and cut her free. Jess was not struggling when viewed from a distance upon first discovery. Her behavioural restriction was assigned an overall grade of “D_2_”, *severe* (Table 2; [18]).

#### Domain 5: Mental State–the Affective Outcomes

*Nutrition.* As Jess worked actively during much of the day, was last fed at least 20 h before her entanglement, and had no access to water whilst caught, she probably experienced significant hunger and thirst, so the related compromise was graded overall as “C_2_”, *moderate* (Table 2; [18]).

*Environment.* Jess’s thermal environment was not particularly challenging, representing *mild* cold exposure, graded “B” (Table 2; [18]).

*Health.* The predominant affect experienced by Jess, evaluated in this domain, would have been pain due to the severe traumatic injury [50,51,52]. Her prioritising of avoidance and escape behaviours is evidence of Jess’s strong focus on her pain experience [51,53]. Although pain would have dominated, Jess was also likely to have been physically exhausted both from her work on the farm and as a result of her efforts to extricate herself from the barbed wire, the combination of the associated affects was judged here to represent *marked* compromise and graded “D_1_” (Table 2; [18]). Once freed, her pain would have persisted at a significant level. Although no longer due to direct stimulation of nociceptors by the wire, voluntary or involuntary movements of her hind limb would likely have added bursts of sharp pain to ongoing marked aching pain [54].

*Behaviour.* Once entangled and during her fruitless attempts to escape, Jess likely experienced various combinations of panic, fear, helplessness and frustration [7,18,33], interacting with the inescapable pain [55], and representing *severe* compromise, graded “D_2_” (Table 2; [18]). Being freed and reassured by the farmer may have had calming effects, but persistent pain and memory of her entanglement would likely have sustained high levels of apprehension whilst in transit to the veterinary clinic, still graded “D_2_” for *severe* compromise (Table 2; [1]).

*Affective contributions to the overall welfare grade.* The combined negative affects of pain, panic, fear, helplessness and frustration were judged to have been overwhelming, leading to an absence of behaviours that reflected enhancement of affective experiences of any type [1]; all domains were therefore graded “0” in this respect (Table 2). The contributory affects in each domain were therefore graded as follows: nutrition “C_2_/0”, environment “B/0”, health “D_1_/0” and behaviour “D_2_/0”. Accordingly, Jess’s overall mental state during this stage was graded “D_2_/0”, i.e., *severe* compromise with *no* enhancement (Table 3).

### 4.3. Stage 3: Veterinary Examination

This stage incorporates Jess’s arrival at the clinic, the initial veterinary examination and her overnight hospitalisation (Table 1). As detailed below, Jess’s overall affective experience, i.e., her welfare status, during this stage was graded “C_2_/0”, i.e., *moderate* compromise with *no* enhancement.

#### Domain 1: Nutrition

Before being offered food and water in the clinic Jess’s nutritional state would have remained at grade “C_2_”, as for stage 2. As she ate all the food offered once kennelled, there would then have been some improvement in her energy-rich nutrient absorption and hydration status. However, this is not likely to have reduced nutritional compromise from grade “C_2_” (*moderate*) to “C_1_” (*mild*), nor to “B” (*low*) (Table 2; [18]), because Jess was offered only a small amount of food in order to decrease the risk of gastrointestinal signs, such as nausea and vomiting, whilst receiving morphine [56].

#### Domain 2: Environment

When in the clinic, during the examination and her subsequent overnight hospitalisation, Jess was in a thermally benign, yet unfamiliar, physical environment, exposed to strange smells, hard surfaces and a cramped kennel, necessitating some adjustment. Taken together, the associated physical/functional compromise was judged to merit a grade of “B”, *low* (Table 2; [18]).

#### Domain 3: Health

Upon arrival at about 6.00 p.m., Jess was not weight bearing on her left hind limb and was given morphine sulphate intramuscularly. The expected time of onset of analgesia was 15–20 min and the duration of action about 3–4 h [56,57]. Recognised potential side effects of opioid use in dogs include sedation, hypothermia, panting, miosis, and more commonly, nausea and vomiting [52,56]. However, opioid-induced vomiting is rare in patients experiencing pain or in the immediate post-operative period. Higher doses can also cause bradycardia and respiratory depression [56]. These side effects were not apparent in Jess, probably because she was a fit and otherwise healthy dog experiencing a significant level of pain. She received a second dose of morphine at about 11.00 p.m., the analgesic effects of which would have worn off approximately 4 h later when she received a third dose [56,57]. Throughout this stage, Jess’s physical injury effectively remained untreated, the additional minimal actions of bandaging her wound and antibiotic administration having little impact on its disabling effects. Overall, therefore, the physical/functional compromise to her health during this stage was graded “C_2_”, *moderate* (Table 2; [18]).

#### Domain 4: Behaviour

Jess was restricted behaviourally throughout this stage because of the injury itself, manual restraint during the clinical examination, close confinement in the hospital kennel and, finally, by the presence of an Elizabethan collar fitted to limit licking or biting behaviour directed at the injury. Added to these physical restrictions were features of the unfamiliar clinic environment, including the presence of strange smells, sounds, animals and people [58]. Jess probably slept intermittently as a result of physical exhaustion and the analgesic and sedative actions of morphine [56]. Overall, the aligned negative behavioural impacts during this stage were judged to be *moderate*, graded “C_2_” on the compromise scale (Table 2; [18]).

#### Domain 5: Mental State—the Affective Outcomes

*Nutrition.* Jess probably continued to experience hunger and thirst until offered food and water in the clinic about 26 h after her last meal on the farm. This, acting together with the sedative effects of morphine, may have reduced the intensity of these affects from *mild* (grade “C_1_”) to *low* (grade “B”). Nevertheless, in accord with the principle of assigning a grade that reflects the most severe compromise in a particular situation in order not to underestimate it [12], the aligned affective experiences were graded “C_1_”, *mild* (Table 2; [18]).

*Environment.* As the thermal environment was benign and the physical discomfort of the hard surfaces and cramped kennel would have been trivial compared to the negative affects captured by considerations related to Domains 3 and 4, the compromise grade assigned here was “B”, *low* (Table 2; [18]).

*Health.* Removal from the vehicle, entry into the clinic and handling at the beginning of the veterinary evaluation probably increased the intensity of Jess’s pain experience. However, the moderately fast-acting analgesic effect of morphine would have provided Jess with some relief which probably continued, due to the second dose given at 11.00 p.m., and a third at 3.00 a.m. [56]. As she was not observed overnight, it is possible that between these administration times she might again have experienced significant pain. During this stage, her pain was judged overall to have been *moderate* and was graded “C_2_” (Table 2; [18]).

*Behaviour.* Jess likely experienced various combinations of fear, anxiety, helplessness and loneliness [7,18,33] due to the challenging novelty of the clinical procedures and environment [59,60], experiences that were potentially exacerbated by, and exacerbated, her pain when that was significant [55]. As with her pain, however, these other experiences would also have been mitigated somewhat by exhaustion-induced and/or morphine-induced sleep and sedation [56], so that, overall for this stage, they were graded “C_2_”, *moderate* (Table 2; [18]).

*Affective contributions to the overall welfare grade.* The combined negative affects of pain, fear, helplessness and loneliness were judged to have predominated during this stage, leading to an absence of behaviours that reflected enhancement of affective experiences of any type; all domains were therefore graded “0” in this respect (Table 2; [1]). The contributory affects in each domain were therefore graded as follows: nutrition “C_1_/0”, environment “B/0”, health “C_2_/0” and behaviour “C_2_/0”. Accordingly, Jess’s overall mental state during this stage was graded “C_2_/0”, i.e., *moderate* compromise with *no* enhancement (Table 3).

### 4.4. Stage 4: Surgical Amputation and Recovery

This stage includes Jess’s preparation for anaesthesia and surgery, the surgery itself, the immediate post-operative recovery period and her progress during the subsequent 28 h (Table 1). The analysis of this stage was conducted in the same way as for stages 1–3. Thus, the two steps of first identifying salient internal physical/functional states and external circumstances, and secondly, considering the potential negative or positive affects that these internal states and external circumstances may generate, were also undertaken here. However, the detailed examples provided for stages 1–3 now allow the sources and the aligned affective outcomes to be combined in a briefer explanation of this stage.

#### 4.4.1. Stage 4a: Preparation for Anaesthesia and Surgery

This covers the pre-operative period until Jess was anaesthetised to surgical depth in preparation for the amputation to begin (Table 1). The overall grade assigned for the first part of this stage was “C_2_/0”, *moderate* compromise with *no* enhancement (Table 3).

*Nutrition.* In view of the small amount of food provided during the previous 36 h and imposed pre-surgical fasting [56,61], Jess’s likely experience of hunger was graded “C_2_”, *moderate* [18], being somewhat blunted by sleep and/or sedation after the fourth dose of morphine followed by acepromazine (Table 1).

*Environment.* Jess’s thermal discomfort was graded “A”, *none*, in view of her thermoneutral physical environment (Table 2; [18]).

*Health.* Preoperative injury-induced pain, blunted first by morphine given at 7.00 a.m. and then by the sedative action of the acepromazine administered at about 10.00 a.m., was graded “B”, *low* (Table 2; [18]).

*Behaviour.* Affects identified as likely in this domain included fear, exacerbated by Jess not being able to predict beneficial outcomes of threatening veterinary manipulations including injections and catheter placement [62,63], and indicated by dilated pupils, crouching with a lowered tail, mouth licking and panting [59,60]. Also likely were helplessness, anxiety, and neophobia [18,33,62]. The compromise associated with these affects was graded “C_2_”, *moderate* (Table 2; [18]).

*Overall affective state.* Jess’s overall negative state was probably dominated by the interacting elements of pain, fear, helplessness, anxiety and neophobia, albeit somewhat blunted by morphine and acepromazine sedation. Nevertheless, their negative impact was still graded “C_2_”, *moderate*, and judged to be sufficient to preclude enhancement in any domain, graded “0” (Table 2; [1]). The combined grade assigned was therefore “C_2_/0” (Table 3).

#### 4.4.2. Surgery

It is self-evident that for an animal to experience anything, negative or positive, it must be conscious. Accordingly, throughout the period of general anaesthesia from induction to its withdrawal once the amputation was complete, Jess was not able to consciously experience anything. Thus, assigning a welfare compromise/enhancement grade was not applicable.

Note, however, that tissue damage caused by and precautions taken during surgery have postoperative impacts that may reduce or increase the intensity/duration of negative affects experienced once consciousness returns. This is illustrated as follows. Although the mid-shaft femoral amputation employed here is often regarded as easier to perform than coxofemoral disarticulation [64], the latter may cause less immediate postoperative pain [65]. Also, epidural administration of bupivacaine plus morphine provides analgesia for 8–24 h and was used here to facilitate the management of immediate and subsequent postoperative pain [56,66] .

#### 4.4.3. Stage 4b: Postoperative Recovery to 28 h

This covers the postoperative period of hospitalisation until Jess was discharged from the clinic the following day. Her overall welfare status for this period was graded “C_2_/0”, *moderate* compromise with *no* enhancement (Table 3).

*Nutrition.* Once conscious and able to stand upright, Jess was offered food. She ate it immediately, indicating persistent hunger, which was not apparently negated by any emetic effects of the morphine. Her hunger was graded overall as “B”, *low* (Table 2; [18]), because of the reasonable amount of food consumed.

*Environment.* Although mildly hypothermic at the end of surgery, Jess was actively warmed until her temperature had returned to the normal range, after which she was held in thermoneutral conditions. The combined affects associated with initial mild thermal discomfort during hypothermia and its subsequent resolution when normothermia was restored were graded “B”, i.e., *low* compromise (Table 2; [18]).

*Health.* During the period immediately after extubation, Jess was not likely to have experienced any postoperative pain or functional impairment due to limb amputation. However, once the effects of the gaseous anaesthesia began to wane she probably became conscious of a loss of sensation in her lower body due to continuing effects of the bupivacaine plus morphine epidural [56,67]. Thus, although relatively pain free, she may well have experienced debility or weakness. The anaesthetic may also have caused nausea and dizziness [68,69]. Towards the end of the anticipated 8–24 h period of analgesia provided by the epidural [56,66], any such debility/weakness had probably waned. Importantly, however, her guarding, restlessness and increased attention to the operative site observed at 11.00 p.m. before receiving the fifth dose of morphine, and again the following morning at 7.00 a.m. when she received her sixth dose, indicated, on both occasions, that she was then experiencing moderate pain [53,56]. This is consistent with the anticipated 3–4 hour duration of analgesia provided by intramuscular morphine [55,57]. Jess subsequently received two further doses of morphine to effect, the first at 11.00 a.m. and the second prior to discharge at 3.00 p.m. Accordingly, the overall impact of these negative affects throughout this period, allowing for transient mitigating effects of morphine overnight, was graded as “C_2_”, *moderate* (Table 2; [18]).

*Behaviour.* Jess was not toileted until the day after surgery, despite fluid therapy during it. This was because of impaired mobility due to the amputation and, initially, to the continuing epidural anaesthesia hindering use of her remaining hind limb [56,67]. Accordingly, Jess was forced to urinate on her bedding, which was probably preceded by significant discomfort of an overfull bladder. Moreover, any subsequent relief was likely to have been counterbalanced by helplessness and discomfort at not being able to avoid contact with the urine in her kennel. The following morning Jess was carried outside for toileting and a brief period of exercise. By avoiding the linoleum flooring in the clinic, this enabled her first postoperative walking experience to be on surfaces allowing appropriate traction. Squatting to urinate, common to female dogs, was difficult for Jess and required support from a sling (Table 1), the unpleasantness of which may have delayed micturition [70]. Overall, the negative affects associated with these behavioural restrictions were graded “C_2_”, *moderate* (Table 2; [18]).

*Overall affective state.* Jess’s overall negative state was probably dominated by the interacting elements of debility/weakness, pain, fear, bladder discomfort, helplessness and frustration, albeit somewhat blunted by morphine sedation. Nevertheless, their overall negative impact was still graded “C_2_”, *moderate*, and judged to be sufficient to preclude behaviours that reflected enhancement of affective experiences of any type; all domains were therefore graded “0” in this respect (Table 2; [1]). The combined grade assigned was therefore “C_2_/0” (Table 3).

### 4.5. Stage 5: Recuperation in a New Home

This stage covers Jess’s recuperation in and adjustment to her new home during the 4 weeks following the amputation. As with the account of stage 4, examples provided by the full two-step welfare analyses conducted to identify internal/external sources of negative or positive affects experienced by Jess during stages 1–3 also allowed a briefer explanation of the grading for this stage. Her overall welfare status during most of this stage was graded “B/+”, *low* compromise with *low-level* enhancement (Table 2), but during the first 7–10 days in particular a grade of “C_1_/+”, *mild* compromise with *low-level* enhancement, was assigned (Table 2).

*Nutrition.* As Jess was offered an optimal amount of high quality dog food and various cooked meats twice daily and had continuous access to fresh water during this stage, hunger and thirst would have been minimal. Also, she likely experienced pleasures associated with eating tasty food and drinking water, as well as comfort due to satiety and good hydration. A grade of “A/+”, *no* compromise with *low-level* enhancement, was therefore assigned (Table 2; [1,18]).

*Environment.* Jess’s cushioned bedding and shelter in the garage afforded her both physical and thermal comfort thereby avoiding compromise in these respects, but there were no welfare enhancing features to this garage environment, therefore attracting a grade of “A/0” for the first 7–10 days of this stage (Table 2; [1,18]). Thereafter, when she could spend more time outdoors during the day, she benefitted from a more varied and benign physical environment, leading to an upgrading to “A/+”, *no* compromise with *low-level* enhancement (Table 2; [1]).

*Health.* About 3 h after her arrival Jess probably experienced significant pain when the analgesic actions of the last morphine dose wore off. During the subsequent 7 days the pain would have lessened progressively, aided by the twice-daily NSAID tablets given in her food. Her wound healing was well advanced by 10 days when the sutures were removed. Likewise, there was a parallel improvement in her capacity to urinate unaided. Moreover, as is commonly observed [71], she was well adapted to walking on three limbs after about 4 weeks. Although the grade assigned for the first 2–3 days was “C_2_/0”, *moderate* compromise with *no* enhancement, overall for this whole period the grade assigned was “B/0”, *low* compromise with *no* enhancement (Table 2; [1,18]).

*Behaviour.* Being mostly confined alone in the unfamiliar and barren garage environment, with outdoor access limited to assisted toileting, the Elizabethan collar preventing her from physically attending to the operative site, and only intermittent contact with her new owners, Jess likely experienced moderate levels of anxiety, neophobia, loneliness, boredom and frustration, indicated during the first 7–10 days by her very unsettled behaviour and repeated attempts to remove the collar. The negative affective consequences of these restrictions were therefore graded “C_2_”, *moderate* compromise (Table 2; [18]), and, because of low motivation and the virtual absence of opportunities to engage in rewarding behaviours in the garage, enhancement was graded “0” (Table 2; [1]). Following suture removal, however, Jess had more freedom to explore outdoors and interact with the Fox Terrier and children, albeit while kept on a lead. These activities, as well as removal of the collar, her progressively improving agility on three legs, and putting Jess in the 50 square metre enclosure during the day while the owners were at work, significantly improved her opportunities to engage in pleasurable behaviours. Access within the enclosure to toys and the other dog for play, bonding and companionship, and a comfortable and sheltered resting area, were regarded as particularly important. Accordingly, the compromise grade was reduced to “B”, *low* [18], and enhancement upgraded to “++”, *mid-level* (Table 2; [1]), for this latter period. The overall grading assigned to behaviour-based affects for this 4-week recuperation and adjustment period was therefore “C_1_/+”, *mild* compromise with *low-level* enhancement (Table 2).

*Overall affective state.* The negatives experiences of pain, anxiety, neophobia, loneliness, boredom and frustration, due to the restrictions that predominated during the first week of this stage, were counterbalanced subsequently by positive experiences linked to Jess’s improving physical agility, greater access to and familiarity with the outdoor environment of her new home, and to diverting interactions with the other dog and children. Throughout she was fed well, was sheltered and had a comfortable resting area. Thus, the contributory grades for affects were: nutrition “A/+”, environment “A/+”, health “B/0”, and behaviour “C_1_/+”. Accordingly, the overall affective grading for this stage was “C_1_/+”, *mild* compromise with *low-level* enhancement (Table 3; [1]).

### 4.6. Stage 6: Subsequent Life as an Amputee

This final stage includes Jess’s life once she had settled into her new lifestyle during her first year as an amputee and pet (Table 1). Although her welfare status, evaluated affectively, would be expected to vary over time, for most of this period it was graded “A/+++”, *no* compromise and *high-level* enhancement (Table 3).

*Nutrition.* As with stage 5, Jess’s food and water intakes continued to be optimal, minimising her experiences of hunger and thirst, continuing to provide her with eating and drinking pleasures and enabling her to gain comfort from an acceptable improvement in her body condition score and muscling to an average-to-good 3/5 (Table 1). She was again assigned a grade of “A/+”, *no* compromise with *low-level* enhancement (Table 2; [1,18]).

*Environment.* As Jess had access to shade/shelter during the day and spent nights in the home, had thick bedding and other soft surfaces in her resting areas and accessed fresh air in her rural environment, she experienced thermal, physical and olfactory comfort and the pleasures of environmental variation. The salient affective outcomes were therefore graded “A/++”, *no* compromise with *mid-level* enhancement (Table 2; [1,18]).

*Health.* As Jess’s exercise levels and food intake maintained her in a good body condition and she had regular health checks, she would have experienced the pleasures of being physically fit and healthy, operating without difficulty within the agility constraints imposed by her amputation [71] and exposed to minimal risk of obesity-induced arthritis in her intact hind leg in later life [72]. She exhibited no extra attention to her amputated limb, so she was not likely to have experienced pain or other unpleasant sensations in the stump or a phantom limb, common in a high proportion of human amputees [73]. The salient affective health outcomes were therefore graded “A/++”, *no* compromise with *mid-level* enhancement (Table 2; [1,18]).

*Behaviour.* Despite her amputation, Jess was capable of engaging in a wide range of rewarding behaviours [1]. The companionship of the Fox Terrier during the day and her owners and their children in the evenings and at weekends provided her with opportunities for play, bonded affection and pleasurable exploratory activities when accompanying her owners as they walked or worked outdoors on their property. She also gave expression to her genetically pre-programmed or learnt stalking and rounding up behaviour when herding sheep, controlled by her owners to remain within her reduced physical capabilities. Thus, once Jess established her new routine she was judged to have utilised enhancement opportunities at a high level (+++). Moreover, as she had the company of the Fox Terrier and toys while restricted to the 50-metre square enclosure during her owners’ absences for work on weekdays, this was not considered to impose significant compromise. Her salient affective behavioural status was therefore graded as “A/+++”, *no* compromise with *high-level* enhancement (Table 2; [1,18]).

*Overall affective state.* The contributory grades for affects during this sixth and last stage were all positive, being: nutrition “A/+”, environment “A/++”, health “A/++” and behaviour “A/+++”. Overall, therefore, the affective grading for this stage was “A/+++”, *no* compromise with *high-level* enhancement (Table 3).

## 5. Discussion

The present Fives Domains assessment and grading of welfare compromise and/or enhancement before, during and after a serious injury to a working farm dog serves several major purposes. First, it illustrates how the structured, systematic and comprehensive analysis achieved by applying the Model can draw attention to areas of animal welfare concern, and importantly, to how welfare enhancement may be impeded or facilitated. The Model therefore supports the exercise of scientifically informed best judgement, which is the hallmark of accomplished animal welfare assessments [12,18]. Second, it shows how the welfare implications of a sequence of events can be traced and evaluated, and in doing so provides specific examples of how the degrees of welfare compromise and enhancement may be graded. These examples are anticipated to assist others to conduct such welfare assessments in other contexts. Third, by assessing an individual dog’s welfare over an extended timescale (stages 1 and 6 are pertinent examples) and by including opportunities for welfare enhancement [1,4] in order to emphasise the balance between positive and negative affective experiences [38], the Five Domain model as used here reinforces the notion that animal welfare status and quality of life are synonymous concepts [6,74,75,76]. And finally, the choice of a companion animal, contrasting its welfare status as a working dog and pet, and considering its treatment in a veterinary clinical setting, help to draw attention to the welfare impacts of various practices. Note, however, that the key features of this fictitious scenario were chosen to highlight use of the Model to identify particular welfare impacts and that, although realistic in some respects, not all working farm dogs and veterinary treatment of major injuries are managed as described here.

### 5.1. Interactions between Welfare Compromise and Enhancement

The updated Model incorporates recognition that the higher the level of compromise in Domains 1–4 the lower will be the motivation for animals to engage in welfare enhancing behaviours [1]. This relates to increases in imbalances or disturbances in the *survival-critical* nutrition, environment and health Domains (1–3) and the intensity of their attendant negative affects in the mental Domain (5). It likewise relates to significant *situation-related* physical restrictions or perceived external threats assessed via the behavioural Domain 4 and the intensity of the associated negative affects (Domain 5). The matrix in Table 2 illustrates this conceptually [1], and stages 2 to 4 of the scenario (Table 1) provide explicit examples of various combinations of pain, panic, fear, frustration, helplessness, loneliness, anxiety and neophobia that were judged to be sufficiently overwhelming to explain an absence of behaviours indicating enhancement of affective experiences of any type [1]. In contrast, stage 6 illustrates that high-level welfare enhancement (“+++” in Domain 5) is promoted when little or no compromise (“A”) is associated with various low-level (“+”) to mid-level (“++”) pleasurable experiences in Domains 1–3, combined with high-level (“+++”) opportunities to engage in rewarding behaviours in Domain 4 (Table 3).

### 5.2. Grading that Accommodates Serial or Oscillating Changes in Circumstances

Two approaches to grading via the Model are apparent. The first approach deals with evaluating serial changes in welfare state. This is illustrated first by the assessment of each stage of the scenario, resulting in grades of “C_1_/+”, “D_2_/0”, “C_2_/0”, “C_2_/0”, “C_2_/0”, “C_1_/+” and “A/+++” being assigned to stages 1–3, 4a, 4b, 5 and 6, respectively (Table 3). This approach is also illustrated by stage 5, where compromise, especially regarding affects associated with the health and behaviour domains, was judged to be greater during the first week after arrival at the lifestyle block than during the subsequent three weeks. In this case, however, the grade assigned for this whole stage was “C_1_/+”, *mild* compromise and *low-level* enhancement. This was determined by offsetting earlier *moderate* welfare compromise and *no* enhancement, graded “C_2_/0”, against the later longer lasting welfare improvements, graded “B/++”, *low* compromise and *mid-level* enhancement.

The second approach to grading accommodates repeated oscillations between two situations where the welfare states differ, so that the impacts of the two need to be balanced in order to assign an overall welfare grade. This is illustrated by stage 1 during which opportunities to engage in rewarding behaviours were severely restricted by kennelling, on average, for 70% of the whole day, but where such opportunities were far greater during farm work for the remaining 30% of the day (equivalent to 60% of daylight hours). The assigned overall grade for this stage was “C_1_/+”, *mild* compromise with *low-level* enhancement (Table 3). Note that the overall grade assigned would have been “C_2_/0”, *moderate* compromise with *no* enhancement had kennelling occupied 80% of the whole day (i.e., farm work occupied 40% of daylight hours), “D_1_/0”, *marked* compromise with *no* enhancement had it occupied 90% of the whole day (i.e., farm work occupied 20% of daylight hours), and “D_2_/0”, *severe* compromise with *no* enhancement had kennelling been continuous with no respite. There are clear implications of these observations for on-farm kennelling of dogs [43], as also for the welfare of dogs confined for long periods in pounds, boarding kennels and shelters [42,77,78].

### 5.3. Assessment of Clinical Procedures

Although several of the interventions undertaken in stages 3 and 4 of the present fictitious scenario might not be in common use, they have been included to allow their potential welfare impacts to be evaluated. Of particular interest are issues associated with pre- and postoperative pain management, especially current confusion regarding the efficacy of analgesic therapies. The reported duration of analgesia for morphine sulphate administered intra-muscularly ranges from 2 to 6 h [79]. Some authors report durations of 3 to 4 h [56,80], whereas others advise an administration frequency of four-hourly [57,81] or claim efficacy exists for “four or more hours” (Epstein [82], p. 174). Also, the wide 12 to 24 h range conventionally reported for epidural morphine [56,66] and the impact of variable levels of pain on its duration of action [56], suggest the need for close monitoring of hospital patients [80].

In Jess’s case, she was left unattended for most of her first postoperative night in hospital during stage 4b. Her behaviour indicated that she was experiencing significant pain before morphine was injected at 11.00 p.m. and again at 7.00 a.m. the next day. However, it is not clear how long she had been in pain prior to these injections. More frequent overnight monitoring and morphine injections could have improved her pain management and welfare status. Alternative pain management protocols might have included constant rate infusion protocols or intermittent doses of epidural analgesia delivered via an indwelling catheter [81,83,84], but all such techniques are only as effective as frequent pain monitoring might allow. Although the scenario presents a striking example of ineffective pain management during this stage, these features were chosen to highlight the importance of frequent evaluations of pain throughout each day and the need to provide analgesic doses to effect rather than rely on conventional reference ranges. Clearly, for such optimisation of analgesic regimes to be effective, veterinary care staff must be able to recognise, interpret and respond to species-specific pain-related behaviours in the clinical setting [58,80].

### 5.4. Other Issues Raised by the Model Analysis

The present analysis has revealed several other significant issues, a detailed consideration of which is beyond the scope of this paper. They are noted here. First, the management of working farm dogs with regard to feeding level and frequency, close confinement in kennels/runs, kennel/run size, shade/shelter, non-work/work exercise opportunities, and opportunities for interactions with people, other dogs and livestock [43]. Second, the therapeutic value of giving equal attention to the affective consequences of both the threatening clinic environment and the traumatic injury being treated, and of recognising that injured dogs may experience several other negative affects in addition to pain [55,85]. Third, the question of whether a prospective Five Domains Model welfare analysis should be made an integral part of decision making about rehoming shelter dogs, both with regard to their suitability to be rehomed at all and the suitability of proposed new homes [86].

### 5.5. Wider Applications of the Model

The present use of the Model draws attention to other more general benefits of such assessments conducted retrospectively or prospectively. For example, informative retrospective welfare analyses such as the one conducted here help to focus attention on areas of concern and to guide the implementation of remedies [1,10], including ways of promoting positive welfare states [4,7,8,39]. In addition, in accord with Canadian experience [87], such retrospective assessments can enhance expert witness participation in successful prosecutions for serious welfare offences by highlighting scientifically supported connections between indicative physical/functional states and behaviours and their associated negative affective experiences in ill-treated animals. Prospectively, the Model is well suited to anticipating the likely good and/or bad welfare impacts of proposed new or modified approaches to managing, housing and interacting with farm [21], zoo [19], research [88] and other animals [18]. Note that its use to prospectively evaluate the impacts of all proposed research, teaching and testing studies on living vertebrates has been a mandatory part of New Zealand’s code of ethical conduct and animal ethics committee system for regulating animal-based science since 1997 [13]. Finally, it is apparent that combining retrospective and prospective analyses to assess an animal’s current and likely future welfare status, i.e., its quality of life [1,6,75,89], could facilitate end-of-life decisions in a wide range of species and circumstances because such Model use makes available helpful information for inclusion in the decision making process.

## 6. Conclusions

This review highlights the structured, systematic and comprehensive approach to animal welfare assessment facilitated by the Five Domains Model. It shows the value of distinguishing between specific internal and external factors and the positive or negative affective experiences they may elicit. It also illustrates the wide range of negative and positive experiences animals, in this example dogs, may have in different circumstances, interactions between those experiences and how they may be graded. In providing insights into the balance between negative and positive experiences, use of the Model provides a broader view of an animal’s welfare status and supports the notion that “animal welfare status” and “quality of life” are synonymous concepts. Finally, it draws attention to the relevance of thorough quality of life assessments to evidence in prosecutions for serious ill treatment of animals, the rehoming of pets and end-of-life decisions.

## Figures and Tables

**Table 1:** Details of the fictitious scenario relating to a working farm dog before, during and after a traumatic injury that required amputation of a hind leg and rehoming to a lifestyle block as a pet.

Stage of the Scenario	Key Features and Events
*1. The working dog prior to the traumatic injury*	Jess, a four-year-old female, was one of four heading dogs used primarily for herding stock on an extensive sheep farm in the North Island of New Zealand. At night and when not working (about 70% of the time), the dogs were kept beside each other in separate raised runs comprising an open-fronted wooden kennel (1 m long, 1 m wide, 1 m high) and a wire mesh enclosed area (2 m × 1 m × 1 m) with wooden flooring. Tarpaulins were placed over the exposed wire area during extreme weather. The dogs were fed supermarket brand dog biscuits and homekill once daily in the evening, with water available in individual two-litre bowls. The dogs were treated for enteric worms and cutaneous fleas during annual vaccination and health checks at the local veterinary practice. Jess was in poor body condition, including a below average skeletal muscle mass, with a score of 2/5.
*2. The traumatic injury*	In mid-May, Jess and the three other dogs were shifting stock. Towards the end of the day Jess sought to retrieve several sheep from over a rise and did not return. Eventually she was found with her left hind leg, immediately below the hock joint, entangled in barbed wire. Her struggling to free herself had resulted in serious injuries to the affected limb. Using wire cutters from a toolbox on his quad bike the farmer cut Jess free. She struggled throughout the few minutes it took to do this. She had a large open wound to the back of her left hock and her foot was becoming swollen. While holding Jess on his farm bike, the farmer drove over the uneven raceway for 30 min back to the farmhouse. After arranging for Jess to be seen by his local veterinarian, he put her into the back of his enclosed utility vehicle and drove for 30 min to the veterinary clinic in town, arriving at about 6.00 p.m.
*3. Veterinary examination*	The veterinarian found Jess’s heart and respiratory rates were moderately elevated, but apart from a low body condition score, including reduced muscling, she presented as a fit, healthy, younger dog with a wound to her left hind limb. Jess was given a moderate–high dose of morphine intramuscularly. She was not weight bearing on her left leg, having a lameness score 10/10. The laceration was full-thickness, and the left common calcanean tendon on the lower limb had been completely severed. Treatment options included surgery to suture the tendon, surgery to amputate the limb, or euthanasia. The farmer decided on amputation and, given her consequent disability, that Jess would be rehomed on a lifestyle block. Also he requested that costs be minimised. The wound was lightly bandaged and broad-spectrum antibiotic was injected subcutaneously. Jess was led on three legs into an indoor clinic kennel. Water and a small amount of canned food were provided and an Elizabethan collar fitted. When the veterinarian administered another dose of morphine at 11.00 p.m., Jess had consumed the food—a third morphine dose was given at about 3.00 a.m.
*4. Surgical amputation and recovery*	*Pre-surgical preparation and the amputation.* Jess received morphine again at 7.00 a.m. the following morning. Whilst still in her hospital kennel she was sedated with subcutaneous acepromazine to act synergistically with the morphine given earlier. In the treatment room, after 30 min, a catheter was inserted into the cephalic vein of her left forelimb to enable injection of sufficient Propofol for Jess to be intubated and then maintained at surgical depth on gaseous isoflurane anaesthesia. In addition, lumbosacral epidural anaesthesia using morphine and bupivacaine was administered at 11.00 a.m. Her left hind limb was prepared for surgery and a saline drip was connected. The mid-shaft femoral amputation involved transection of the bone and proximal hind limb musculature, and ligation of the associated vasculature and nerves. Sterile technique was employed throughout.
	*The initial recovery period.* Following surgery, the wound was bandaged, the gaseous anaesthesia was discontinued and Jess was placed in her kennel in right lateral recumbency on a heated blanket and covered with other blankets. She was extubated once sufficiently conscious to regain her gag reflex. Her rectal temperature, which had decreased to 36.5 °C during surgery, was monitored until it reached at least 37.5 °C; the heated blanket was then removed. After initial attempts to sit upright, Jess lay on her unaffected side and rested. Offered canned wet food and water in the early afternoon, Jess ate the food vigorously, after which a second dose of broad-spectrum antibiotic was given subcutaneously. The Elizabethan collar was fitted again to reduce the risk of her licking her wound or removing her sutures. She was not toileted outside, as her intact hind limb may still have been affected by the epidural. An additional dose of morphine was given at 11.00 p.m.
	*The subsequent recovery period.* Jess received morphine again at 7.00 a.m. the following day. She was toileted outside, assisted with a soft towel sling under her abdomen because she had difficulty squatting to urinate. Once back in her kennel, she was offered, and vigorously ate, food containing a pill of the non-steroidal anti-inflammatory drug (NSAID) carprofen. Morphine was injected again at 11.00 a.m. To reduce costs to the farmer Jess was discharged at 3.00 p.m. after receiving a final dose of morphine. The discharge instructions were to limit her activity during the first 10 days, but maintain twice to three times daily short toilet walks to encourage the use of her legs. She was prescribed seven days of twice-daily NSAID pain relief and broad-spectrum antibiotic, both to be given in her food. Her sutures were scheduled for removal after 10 days.
*5. Recuperation in a new home*	After 1 h in the farmer’s utility vehicle Jess arrived at the lifestyle block. Owned by a young couple with two children aged 8 and 10 years, the block accommodated a small flock of sheep, several finisher beef cattle, some chickens and a neutered male Fox Terrier dog. Jess was placed in a garage on a small mattress with a bowl of water nearby and was fed wet food and biscuits. She still required some assistance from a towel sling that evening when toileted on the grass outside. The discharge instructions were followed for the next 10 days. The Elizabethan collar was fitted except when she was being fed. Initially she was very unsettled and repeatedly tried to remove the collar. At suture removal after 10 days, her wound had healed well, the swelling was much reduced and she walked unassisted into the veterinary clinic. Thereafter, her activity levels were progressively increased. She was introduced to the Fox Terrier and the children, becoming increasingly at ease with them, and gradually spent time in a 50-metre square fenced pen containing a comfortable kennel.
*6. Subsequent life as an amputee*	About six weeks after the amputation, when supervised, Jess was allowed off lead around the farm. She was also allowed in the house with the family and slept on a luxurious pet bed in the lounge at night. She continued to be offered a variety of premium quality canned dog foods and biscuits twice daily, plus a variety of cooked meats, and was regularly bathed and treated for both fleas and intestinal worms. She now had an average-to-good body condition score of 3/5 with appropriate muscling, could run effectively on three legs, played well with the children, and was occasionally allowed to herd the sheep. Her owners worked in town Monday to Friday between 8.00 a.m. and 6.00 p.m., at which times Jess was placed in the 50-metre square fenced area, with toys for play, a kennel for shelter and fresh water provided. The Fox Terrier had access to the house through a cat flap, but tended to remain outdoors with Jess for companionship.

**Table 2:** A conceptual matrix combining welfare compromise and enhancement grades assigned using the Five Domains Model. It shows that as welfare compromise increases, animals’ motivation to engage in behaviours they may find rewarding decreases, reaching zero at the two highest levels compromise [1]. See text for details.

Welfare Compromise Grade	Welfare Enhancement Grade			
	None 0	Low-level +	Mid-level ++	High-level +++
A None	(A/0) ^(3)^	A/+	A/++	A/+++
B Low	B/0	B/+	B/++	–
C Mild to moderate ^(1)^	C_2_/0	C_1_/+	–	–
D Marked to severe ^(2)^	D_1&2_/0	–	–	–
E Very severe	E/0	–	–	–

^(1)^ Grade “C” has subdivisions, C_1_ and C_2_, representing “mild” and “moderate” compromise, respectively; ^(2)^ Grade “D” has subdivisions, D_1_ and D_2_, representing “marked” and “severe” compromise, respectively; ^(3)^ A theoretical possibility not likely to be encountered in practice because the absence of compromise would require some very low-level enhancement [1].

**Table 3:** Welfare compromise and enhancement grades for the six stages of the scenario (Table 1) using the updated Five Domains Model [1]. See text for details. Grades are assigned for Domains 1–5, the overall welfare state being represented by Domain 5 (mental state). Refer to Table 2 and Figure 1 for details of the grading system.

Stage	Domain				
	1 Nutrition	2 Environment	3 Health	4 Behaviour	5 * Mental State
1 Prior to the traumatic injury	C_1_/0	B/0	B/++	C_1_/+	C_1_/+
2 The traumatic injury	C_2_/0	B/0	D_1_/0	D_2_/0	D_2_/0
3 Veterinary examination	C_1_/0	B/0	C_2_/0	C_2_/0	C_2_/0
4 Surgical amputation and recovery					
*(a) Preparation for anaesthesia and surgery*	C_2_/0	A/0	B/0	C_2_/0	C_2_/0
*(b) Postoperative recovery to 28 h*	B/0	B/0	C_2_/0	C_2_/0	C_2_/0
5 Recuperation in a new home	A/+	A/+	B/0	C_1_/+	C_1_/+
6 Subsequent life as an amputee	A/+	A/++	A/++	A/+++	A/+++

* Overall welfare status. Welfare compromise grades: A None; B Low; C_1_ Mild, C_2_ Moderate; D_1_ Marked, D_2_ Severe; E Very severe. Welfare enhancement grades: 0 None; + Low-level; ++ Mid-level; +++ High-level.
